# Comprehensive characterisation of intronic mis-splicing mutations in human cancers

**DOI:** 10.1038/s41388-020-01614-3

**Published:** 2021-01-08

**Authors:** Hyunchul Jung, Kang Seon Lee, Jung Kyoon Choi

**Affiliations:** 1 Department of Bio and Brain Engineering, KAIST, Daejeon, 34141 Republic of Korea; 2 Cancer Ageing and Somatic Mutation Programme, Wellcome Sanger Institute, Cambridge, UK; 3 Penta Medix Co., Ltd., Seongnam-si, Gyeongi-do 13449 Republic of Korea

**Keywords:** Cancer genomics, RNA splicing

## Abstract

Previous studies studying mis-splicing mutations were based on exome data and thus our current knowledge is largely limited to exons and the canonical splice sites. To comprehensively characterise intronic mis-splicing mutations, we analysed 1134 pan-cancer whole genomes and transcriptomes together with 3022 normal control samples. The ratio-based splicing analysis resulted in 678 somatic intronic mutations, with 46% residing in deep introns. Among the 309 deep intronic single nucleotide variants, 245 altered core splicing codes, with 38% activating cryptic splice sites, 12% activating cryptic polypyrimidine tracts, and 36% and 12% disrupting authentic polypyrimidine tracts and branchpoints, respectively. All the intronic cryptic splice sites were created at pre-existing GT/AG dinucleotides or by GC-to-GT conversion. Notably, 85 deep intronic mutations indicated gain of splicing enhancers or loss of splicing silencers. We found that 64 tumour suppressors were affected by intronic mutations and blood cancers showed higher proportion of deep intronic mutations. In particular, a telomere maintenance gene, *POT1*, was recurrently mis-spliced by deep intronic mutations in blood cancers. We validated a pseudoexon activation involving a splicing silencer in *POT1* by CRISPR/Cas9. Our results shed light on previously unappreciated mechanisms by which noncoding mutations acting on splicing codes in deep introns contribute to tumourigenesis.

## Introduction

Introns account for 42% of the human genome and contain essential splicing codes that control the fidelity of pre-mRNA splicing [1, 2]. Splicing codes mainly consist of core and auxiliary *cis*-splicing elements. The core splicing codes include donor and acceptor splice sites (SSs), branchpoints (BPs), and polypyrimidine tracts (PPTs). Splicing enhancers and silencers compose the auxiliary elements [3]. These codes are recognised by spliceosome to promote a precise excision of introns during pre-mRNA splicing. The donor and acceptor SSs are located at exon-intron junction and mediate recognition by major splicing machinery (e.g., *U1* and *U2AF1*). The BPs are present upstream of the acceptor SSs (i.e., located between 18 and 40 bp upstream from the acceptor SSs) and serve as binding sites for *U2AF1* [4]. The PPTs rich with uracil nucleotides are located between the acceptor SSs and BPs and serve as binding sites for *U2AF2* [5]. The splicing enhancers and silencers located throughout exons and introns stimulate and inhibit splicing by interacting with SR proteins hnRNPs [6], respectively. A growing number of human diseases are being associated with mutations that alter the fidelity of these splicing regulatory codes and lead to mis-splicing such as exon skipping and intron retention [7–9]. For example, 15–50% of disease-causing mutations in monogenic diseases, including those within exons, were estimated to affect pre-mRNA splicing [10, 11].

Previous cancer studies on changes in mRNA splicing were concentrated on alternative splicing [12–14], whereas a relatively small number of studies addressed mis-splicing mutations using large-scale next-generation sequencing data [15–18]. Mis-splicing mutations in tumour suppressor genes (TSGs) frequently introduce premature termination codons (PTCs) in abnormally spliced transcripts and elicit mRNA degradation via nonsense-mediated decay (NMD) [11]. However, these studies were based on exome data and thus our current knowledge is largely limited to exons and the canonical SSs. Recent transcriptomic analyses have identified a number of deep intronic mis-splicing variants in the causal genes of Mendelian disorders [19–22]. These deep intronic mutations frequently induce pseudoexon inclusion by creating denovo SSs, resulting in loss of function of genes by introducing PTCs. Somatic mutations in intronic splicing codes have the potential to affect pre-mRNA splicing in cancer [23, 24]. However, to our knowledge, no comprehensive studies of mis-splicing intornic mutations have not been conducted due to the lack of large-scale matched whole-genome sequencing (WGS) and RNA-seq data. The Pan-Cancer Analysis of Whole Genomes (PCAWG) project produced thousands of WGS data with matched RNA-seq profiles, thereby offering the opportunity for a systematic analysis of intronic mutations that alter mRNA splicing across different types of human cancers. Here, we analysed 1134 tumour samples with WGS and RNA-seq data to identify *cis*-acting somatic mutations associated with abnormal splicing, including those in deep introns.

## Results

### Identification of mis-splicing mutations using read ratios and allele specificity

The ratio-based identification of abnormal splicing, using a background distribution built from ~4200 normal and cancer samples (Supplementary Table 1), resulted in 1627 mutations (1318 intronic and 366 exonic; Supplementary Tables 2–6), including single nucleotide variants (SNVs) (*n* = 1469; 90.1%) and small deletions (*n* = 120; 7.6%) (Supplementary Fig. 1a). A large fraction (*n* = 585; 45.9%) of the identified intronic mutations were located at the SS dinucleotides. Intronic mutations, which were more than 20 bp away from the nearest exon-intron junction, were defined as deep intronic mutations, because the fraction of the mutations discovered by whole-exome sequencing started dramatically declining at 20 bp from the nearest exon-intron junction (Supplementary Fig. 1b). Deep intronic (>20 bp from authentic junctions) and proximal intronic (3–20 bp from authentic junctions) mutations comprised 32.3% (*n* = 309) and 21.8% (*n* = 369), respectively (Supplementary Fig. 1c). Among the exonic variants, 27% (*n* = 99) were silent mutations (Supplementary Fig. 1d), reflecting the role of synonymous mutations in mRNA splicing during tumourigenesis [15, 16].

**Fig. 1:**
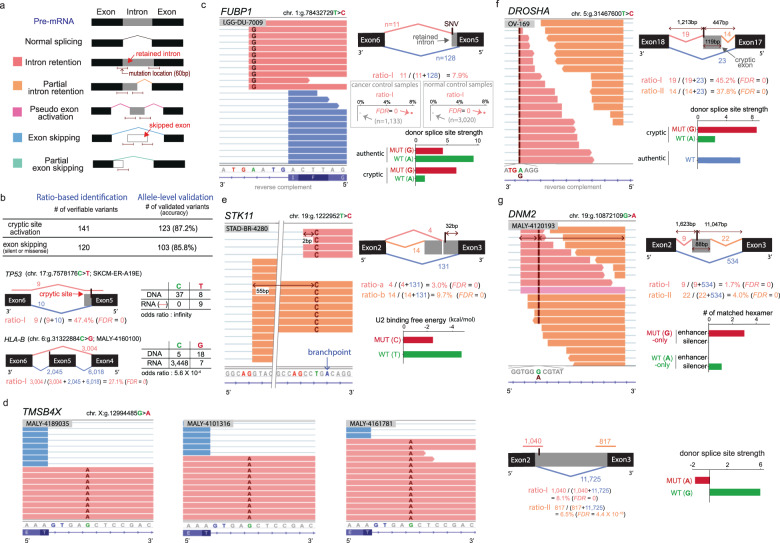
Read ratio-based identification of abnormal splicing and allele-based validation. **a** Schematic representation of different types of aberrant splicing events. **b** Summary and examples of allele-based validation. Among mutations within 30 bp from activated cryptic SSs, those spanned by ≥3 RNA-seq reads were defined as verifiable variants (Supplementary Table7). Allele specificity was assessed by the 2 × 2 contingency table. For example, all RNA-seq reads covering the cryptic SS (*n* = 9, red) of *TP53* carried the mutant allele (T). In addition, exonic mutations within 10 bp from the exon-intron junction of skipped exons were collected as verifiable variants and assessed by the 2 × 2 contingency table. For example, there was a significant underrepresentation of the mutant allele, G, in the RNA-seq reads covering this region of *HLA-B*. **c–g** Read ratios and allelic patterns of mis-splicing examples. In the browser views, the RNA-seq reads supporting abnormal and normal splicing are shown in pink/orange and blue, respectively, with mutations highlighted in dark red. Below, cryptic and authentic SSs are marked in red and blue, respectively, with wild-type sequences shown in green. The schematic illustrations include the number of abnormally (pink/orange) and normally (blue) spliced RNA-seq reads. **c** Proximal intronic mutation causing partial intron retention in *FUBP1*. The proportion of abnormally spliced reads over all reads (ratio-I = 7.9%) was calculated from a lower grade glioma sample (DU-7009). Null distributions of read ratios were derived from 3020 normal and 1133 cancer samples without mutations in *FUBP1*. The observed ratio was then transformed into the Z-score, and its right-tail *P* value was estimated. The strength of the cryptic donor SS was estimated using MAXENT for sequences 3 bp upstream to 6 bp downstream of the cryptic splice site with the mutant and wile-type allele. **d** Recurrent mutations causing full intron retention in *TMSB4X*. The identical proximal intronic mutations were identified in three different malignant lymphoma samples. The results of the ratio-based analysis and SS strength analysis (right) are from MALY-4189035. **e** Deep intronic BP mutation leading to partial intron retention in *STK11* by two different cryptic acceptor SSs, indicated in pink and orange. The cryptic SSs associated with the partial intron retention (ratio-a and -b) are observed at 3 bp and 56 bp upstream of the variant, supported by the red and orange reads, respectively. The U2 binding free energy was calculated for sequences 5 bp upstream to 3 bp downstream of the BP for the mutant and wile-type allele. **f** Deep intronic mutation activating a pseudoexon through the gain of a cryptic donor SS in *DROSHA*. The strength of the cryptic donor SS with the mutant or wild-type allele was compared to the authentic donor SS of exon 17. **g** Deep intronic mutation activating a pseudoexon through the gain of a splicing enhancer in *DNM2*. Sequences ±5 bp from the mutation with the mutant or wild-type allele were matched with known splicing enhancer/silencer motifs.

The abnormal splicing events we identified in association with matched mutations included full or partial intron retention, full or partial exon skipping, pseudoexon activation, and combinatorial abnormal splicing (Fig. 1a and Supplementary Fig. 2). Exon skipping (*n* = 482) was the most frequent type of abnormal splicing, followed by partial intron retention (*n* = 421) and partial exon skipping (*n* = 385). The number of full intron retention cases (*n* = 90) may be underestimated because of more strict detection criteria involving the assessment of allele specificity (Supplementary Fig. 2).

Allele specificity was used for the validation of splicing events (Fig. 1b). For partial intron retention and pseudoexon activation, we assessed whether the RNA-seq reads supported cryptic splice-site activation in an allele-specific manner by using the 2 × 2 contingency table. A majority of verifiable mutations (123 of 141) were overrepresented among the relevant RNA reads (Fig. 1b and Supplementary Table 7). For example, the partial intron retention associated with the splice-site C-to-T mutation in *TP53* was validated by overrepresentation of the T allele among RNA reads spanning the cryptic site (Fig. 1b). For exon skipping, the mutant alleles should be underrepresented in RNA reads covering the skipped exons. Thus, we evaluated enrichment of the exonic mutations associated with exon skipping by the DNA and RNA allele counts. Again, a majority of verifiable exonic mutations (105 of 120) passed this test (Fig. 1b and Supplementary Table 8). For example, the mutant G allele was less frequently observed than expected on the 3′ end of the skipped exon of *HLA-B* (Fig. 1b). These high validation rates support the reliability of the mutations identified by the read-ratio method.

A few examples of the identified splicing alterations are shown in Fig. 1c–g. The partial intron retention detected in *FUBP1* was assessed by the background distribution of read ratios derived from reference samples with wild-type *FUBP1* (Fig. 1c). The mutant allele, G, was observed only on reads covering the retained intronic sequences. Recurrent mutations causing full intron retention were identified in *TMSB4X* (Fig. 1d). The identical proximal intronic mutations were discovered in three different malignant lymphoma samples. The partial intron retention of *STK11* involved a deep intronic mutation that was near the BP and 32 bp away from the exon 3 junction (Fig. 1e). The mutant allele, C, was observed only on reads derived from the two mis-spliced transcripts. Pseudoexon activation by deep intronic mutations was found in *DROSHA* (Fig. 1f), *DNM2* (Fig. 1g), and *POT1* (described later). The mutation in *DROSHA*, the core nuclease that executes microRNA processing, created a cryptic donor SS. The *DNM2* and *POT1* mutations were associated with gain of a splicing enhancer and loss of a splicing silencer. *POT1* mis-splicing was recurrently observed in two tumour samples. In all the three cases, RNA-seq reads covering the cryptic site carried only the mutant allele.

### Characterisation of proximal intronic mis-splicing mutations

**Fig. 2:**
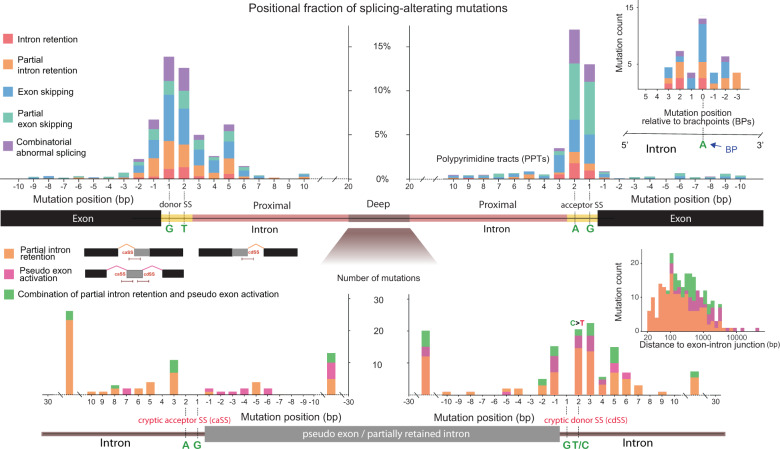
Distribution of mis-splicing mutations near exon-intron junctions. Intronic mutations 1–2 bp, 3–20 bp, and >20 bp away from the nearest exon-intron junction were classified as SS, proximal, and deep intronic mutations, respectively. For deep intronic mutations, we limited our analysis to those that were surrounding BPs (right inset in the top panel) or activated cryptic SSs (bottom panel). Fraction of mis-splicing mutations over all variants found at each position (top panel). The count of mutations near BPs and U2 binding residues (5 bp upstream to 3 bp downstream of the BPs) is shown in the right inset. Distribution of deep intronic mis-splicing mutations (bottom panel) according to the distance from activated cryptic acceptor SSs (caSSs) or cryptic donor SSs (cdSSs). Wild-type dinucleotides at cryptic SSs are shown in green. Mutation counts according to the distance from the nearest authentic exon-intron junction are shown in the right inset.

We examined the locational effect of proximal intronic, SS, and exonic mutations based on the fraction of mis-splicing mutations over all variants at each position (Fig. 2, top panel). The greatest effects were observed around the canonical dinucleotides GT and AG for donor and acceptor SS, respectively [9]. In particular, mutations associated with partial exon skipping were more frequent at the acceptor than at donor SSs (green bars in the top panel of Fig. 2). This is in agreement with the previous report that cryptic acceptors are more common than cryptic donors for partial exon skipping [25].

**Fig. 3:**
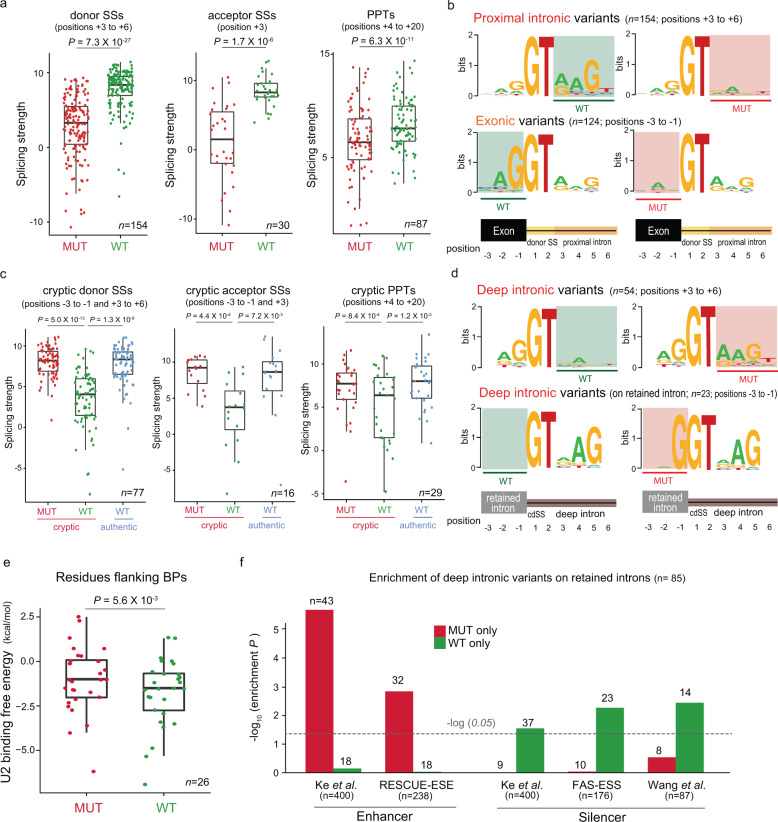
Alterations of splicing codes by intronic mutations. **a** Loss of core splicing codes by proximal intronic mutations. Differences in the strength of SSs or PPTs between the mutant and wild-type allele, as estimated by MAXENT [27]. The Wilcoxon signed-rank test was used to assess the statistical significance. **b** Changes in sequence consensus by proximal intronic or exonic mutations near donor SSs. The consensus motifs were derived from the 9-mers of the authentic donor SSs with the GT dinucleotide at the border. **c** Gain of core splicing codes by deep intronic mutations. Shown are the differences in the strength of SSs or PPTs between the mutant and wild-type allele. Also compared is the strength of the corresponding authentic SSs with the reference sequences. **d** Changes in sequence consensus by deep intronic mutations at cryptic donor SSs. The motifs were derived from the 9-mers of cryptic donor SSs, with the GT dinucleotide at the border. **e** Differences in the U2 binding free energy between the mutant and wild-type allele. The binding free energy of U2 snRNA was calculated for sequences 5 bp upstream to 3 bp downstream of BPs while excluding the BP nucleotide by using the Vienna RNA package [56]. **f** Alterations of auxiliary splicing codes by deep intronic mutations. The nucleotide sequences ±5 bp from the mutation with the mutant or wild-type allele were matched with the known sets of motifs. The number of sequences present in each motif set is shown above the bar, after removing duplicate instances. The *P* values were calculated by random permutation tests.

Flanking sequence mutations were more frequent near donor SSs than acceptor SSs (see the exonic −1 to −3 and intronic +3 to +6 bases in the top panel of Fig. 2). These regions correspond to the binding site of U1 snRNA [26]. While the importance of the −1 nucleotide of donor SSs has previously been reported [15], our results reveal that genetic changes in the intronic sequences flanking the GT splice donor are prone to splicing aberration in human cancers. Indeed, the mis-splicing mutations arising between the +3 to +6 bases were found to weaken the strength of donor SSs (Fig. 3a, left). A similar trend was found for the −1 to −3 bases and donor strength (Supplementary Fig. 3a, left). The *FUBP1* splicing change (Fig. 1c) illustrates a loss of an authentic donor SS and a creation of a cryptic donor SS by the +4 mutation. A similar example was found for *TMSB4X*, in which the loss of the donor SS due to the +5 mutations that are recurrent in three different tumours resulted in full intron retention (Fig. 1d). We observed distinct motifs and their disruption by mutations. The intronic +3 to +6 bases with mis-splicing mutations showed a sequence consensus that was broken by mutations (Fig. 3b, upper). Similarly, the exonic −3 to −1 nucleotides with mis-splicing mutations exhibited consensus only among the wild-type sequences (Fig. 3b, lower). These motifs resemble two typical consensus 5′ splice motifs, that is, right- and left-handed motifs with higher relative entropies in the intron and in the exon, respectively [27, 28].

Regarding the acceptor SSs, mutations at the +3 nucleotide, to which U2AF1 binds [26], showed a high rate of abnormal splicing (Fig. 2, top panel). These mutations were found to significantly lower the strength of the acceptor SSs (Fig. 3a, middle). Mutations between the −1 and −3 bases also negatively affected acceptor strength (Supplementary Fig. 3a, right). The PPTs are essential splicing codes for directing U2AF2 to the acceptor SS [5]. Mutations on the PPTs (positioned between the +4 and +20 bases) significantly lowered the acceptor SS strength (Fig. 3a, right). We observed the disruption of consensus sequences by mutations in the flanking regions of the acceptor SSs (Supplementary Fig. 3b).

Exonic mutations distant from the SS (from positions −4 to −30) showed relatively low rates of abnormal splicing (0.3% on average) (Fig. 2, top panel). However, those associated with exon skipping were accompanied by loss of splicing enhancer motifs (*P* = 7.5 × 10^−4^) and gain of splicing silencer motifs (*P* = 3.8 × 10^−3^) (Supplementary Fig. 3c). Genetic disruption of exonic splicing enhancers or silencers is often observed in human inherited diseases [29].

### Characterisation of deep intronic splicing mutations

Deep intronic mutations may result in partial intron retention or pseudoexon activation (bottom left inset of Fig. 2). In particular, pseudoexon activations can primarily result from deep intronic mutations. These mutations can arise at any residues inside introns (bottom right inset of Fig. 2). Their distance to authentic exon-intron junctions ranged up to 10 kb (mean distance = 784 bp). Mutations relatively close to exon-intron junctions tended to result in partial intron retention while those far from the junctions were more associated with pseudoexon activation (bottom right inset of Fig. 2). Pseudoexons ranged in size from 28 to 880 bp, with an average of 123 bp (Supplementary Fig. 3d). Some mis-splicing events were a mixture of partial intron retention and pseudoexon activation (see Supplementary Fig. 4 for examples).

Proximal intronic mutations (Fig. 2, top panel) disrupt consensus sequences and weaken donor/acceptor strength (Fig. 3a, b and Supplementary Fig. 3a, b). In contrast, deep intronic mutations should create cryptic donor/acceptor SSs to cause partial intron retention or pseudoexon activation. Indeed, deep intronic mutations were concentrated at the 3′ end of pseudoexons or partially retained introns (the bottom panel of Fig. 2). All these mutations occurred near pre-existing GT dinucleotides or converted CT dinucleotides into the canonical GT signal (the bottom panel of Fig. 2 and Supplementary Fig. 5a). Similar to authentic donor SSs (Fig. 2, top panel), the −1, +3, and +5 nucleotides appear to play an important role in cryptic donor SSs (Fig. 2, bottom panel). The mutations around cryptic donor SSs increased splicing strength (Fig. 3c, left). Importantly, the resulting strength of the gained cryptic SSs was comparable to the strength of the matched authentic SSs (Fig. 3c, left). The −1 mutation in *DROSHA* (Fig. 1f) created a cryptic donor SS that was stronger than the authentic site. The changes in consensus sequences flanking cryptic donor SSs were exactly opposite to those flanking authentic donor SSs (compare Fig. 3d and b). Taken together, deep intronic mutations can induce partial intron retention or pseudoexon activation by creating the canonical sequences that can serve as splicing donors.

Whereas the donor mutations were concentrated near the SS, the mutations associated with acceptor gain were scattered across the SS (Fig. 2, bottom panel). All the observed cryptic acceptor SSs were created around pre-existing AG dinucleotides. The importance of the +3 residue, the first intronic sequence following the canonical GT and AG signal, was repeatedly found in all cases (Fig. 2, bottom panel). Mutations at positions from −3 to +3 were found to significantly increase splicing strength (Fig. 3c, middle). In addition, there was a high frequency of mis-splicing mutations between the +10 and +30 residues (Fig. 2, bottom panel), probably reflecting the presence of cryptic PPTs. In fact, in comparison to the wild-type sequences, mutations at positions from +4 to +20, corresponding to the PPT locations, significantly increased splicing strength to a level comparable to the matched authentic acceptor SSs (Fig. 3c, right).

Together with SSs and PPTs, BPs compose core *cis*-splicing elements [7]. U2 snRNA binds BPs and their flanking regions. We identified 53 mutations from regions 5 bp upstream to 3 bp downstream of BPs (Supplementary Fig. 5b). While mutations at BPs displayed the highest mis-splicing rate, the +2 and −2 residues also showed large effects (top right inset of Fig. 2). To understand the effect of U2 snRNA binding disruption, we measured the differences in the U2 binding free energy between the mutant and wild-type allele. The splicing mutations we identified significantly elevated the U2 binding free energy (Fig. 3e), indicating decreased binding stability. In the case of the *STK11* intron retention (Fig. 1e), a deep intronic mutation 2 bp away from the BP increased the U2 binding free energy.

Lastly, we examined the role of the splicing enhancers and silencers in inducing partial intron retention and pseudoexon activation. We performed an enrichment analysis with respect to splicing enhancer and silencer motifs for mutations (*n* = 84) on pseudoexons or partially retained introns except at the −1 to −3 positions. This analysis revealed that the mutation alleles consistently indicated enhancer gain and silencer loss (Fig. 3f). Such examples were found in *DNM2* (Fig. 1g) and *POT1* (described later). As described later, we performed experimental validation of the effect of the silencer loss in *POT1*. Altogether, the gain of the core splice codes and the alteration of the auxiliary splicing elements by deep intronic mutations represent an important, previously unappreciated mechanism of partial intron retention and pseudoexon activation in human cancers.

### Selection on mis-splicing mutations in cancer

**Fig. 4:**
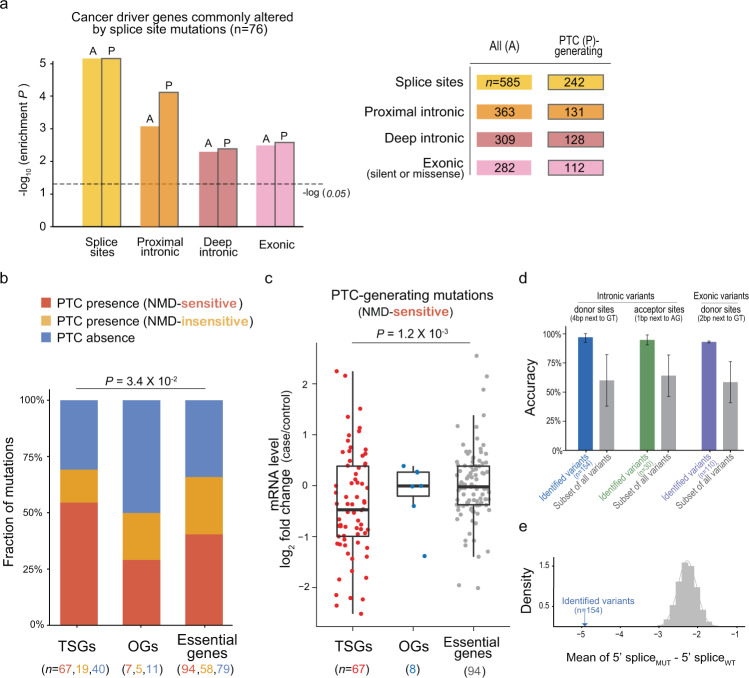
Mis-splicing mutations in tumour suppressor genes and prediction models. **a** Enrichment of the identified mis-splicing mutations in cancer driver genes commonly altered by splice site mutations. We used genes with type ‘S’ (splice site) mutations in the COSMIC census [49]. The *P* values were calculated by random permutation tests. **b** Fraction of PTC-generating mutations in TSGs, oncogenes (OGs), and essential genes [64]. According to the PTC location, the PTC-generating variants were divided into NMD-sensitive and NMD-insensitive groups. The *P* values were assessed using the one-sided Fisher’s exact test on the 2 × 3 contingency table. **c** Effect of NMD-sensitive PTCs on mRNA expression by gene class. For each gene per cancer type, the control expression level was calculated by averaging the gene expression levels of wild-type samples (≥5). The *P* values were calculated using the two-sided Mann–Whitney U test. **d** Performance of the prediction models. Shown are the accuracies for the intronic mutations near the donor SS (blue; +3 to +6) or acceptor SS (green; +3), and exonic mutations flanking the donor SS (purple; −2 to −1) in comparison to the accuracies of the same models with randomly selected mutations at the corresponding SSs (grey). **e** Example of feature value distribution. The density plot shows the mean of feature values (maxent score of 5′splice_MUT_ – 5′splice_WT_) used in the predictive model for intronic mutations near the donor SS. The distribution of expected values was obtained from random mutation selection (100,000 trials).

To investigate the relevance of mis-splicing intronic mutations in tumourigenesis, we first examined whether the intronic mutations were enriched in the cancer driver genes associated with reported SS mutations. Overrepresentation of exonic (silent or missense) mis-splicing mutations in this gene set was previously reported [15]. Here, we found that these genes undergo abnormal splicing by deep intronic mutations to a similar extent as exonic mutations (Fig. 4a). The intronic mutations as well as the SS and exonic mutations were overrepresented in TSGs but not in oncogenes (Supplementary Fig. 6), implying that many splicing alterations result in a functional disruption that is subject to selection. Loss-of-function mis-splicing mutations can act by generating a PTC that triggers NMD [30, 31]. Although the proportion of PTC mutations was similar between essential genes and TSGs, there was a significant difference in the fraction of NMD-sensitive and NMD-insensitive PTCs (Fig. 4b; *P* = 3.4 × 10^−2^). Because essential genes need to be expressed at a high level, NMD-sensitive mutations in these genes may undergo negative selection. In contrast, the high fraction of NMD-sensitive PTCs in TSGs may indicate positive selection. NMD of TSGs should be favoured in cancer evolution because NMD can lower the expression level of transcripts that remain partly or fully functional despite PTCs. In fact, mis-splicing mutations leading to NMD-sensitive PTCs in TSGs significantly lowered gene expression (Fig. 4c). This pattern was not observed for essential genes and oncogenes, probably reflecting a transcriptional compensation mechanism.

As shown above, some mis-splicing mutations, especially those in TSGs, may lower the overall expression levels via NMD. If the expression level is below the detectable range of RNA-seq, our read ratio-based method may not be able to detect such mis-splicing mutations. In addition, we used rigorous criteria to detect mis-splicing mutations, likely resulting in some false negatives. For example, our method excluded the cases in which there are additional mutations in the given gene. These called for prediction models that only use mutation patterns independently of RNA read counts. Thus, we built three classification models that consider proximal intronic mutations at the locations with a high fraction of abnormal splicing (>2%), that is, intronic donor sites (+3 to +6), intronic acceptor sites (+3), and exonic donor sites (−2 to −1). Based on a one-class supporter vector machine, each model was trained with splicing strength changes computed from the identified splicing mutations (Supplementary Table 9). The threefold cross-validation (1000 iterations) of the models showed a high mean accuracy of 93–97% (Fig. 4d, e and Supplementary Fig. 7). The models that were trained on the same number of randomly selected mutations at the corresponding sites showed a significantly lower average accuracy of 59–64% (Fig. 4d and e). We predicted 2867 variants to be able to alter RNA splicing in cancer. Among them, 115 were in TSGs (Supplementary Table 10).

### Intronic mis-splicing mutations in cancer genes across cancer types

**Fig. 5:**
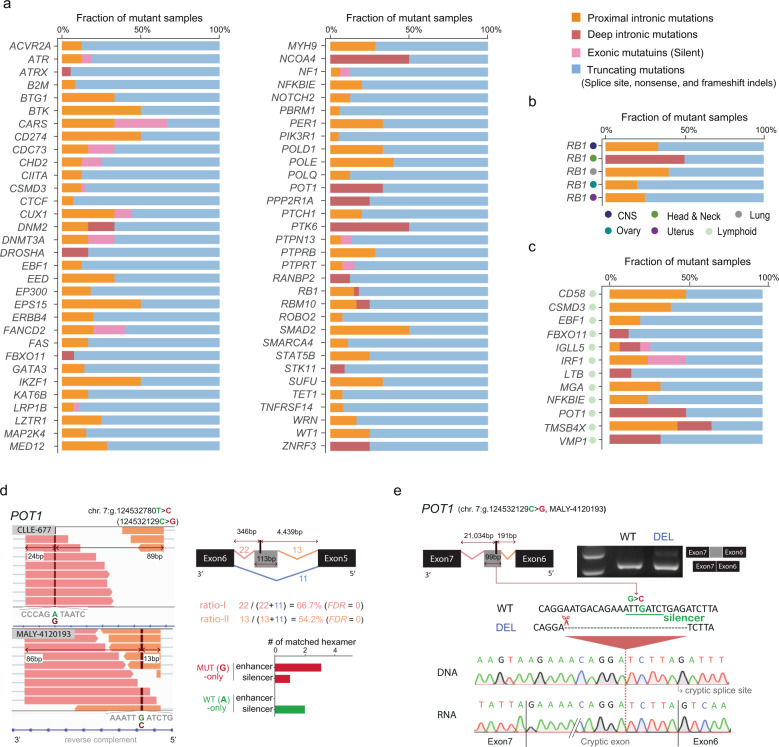
Relevance of mis-splicing mutations in tumourigenesis. **a** Proportion of samples with mis-splicing mutations compared with those having traditional truncating mutations. Known tumour suppressor genes for which intronic mutations (proximal and deep) account for >5% of the samples are shown. The samples with predicted proximal and exonic mutations were included. **b** Proportion of mutant samples in *RB1* per tissue type. **c** Proportion of mutant samples in lymphoid tissue. Potential tumour suppressor genes were included. **d** Deep intronic mutations leading to pseudoexon activation in *POT1*. The results of the read ratio analysis and motif analysis are for the event identified in a chronic lymphocytic leukaemia sample (CLLE-677). Sequences ±5 bp from the mutation with the mutant or wild-type allele were matched with known splicing enhancer/silencer motifs. **e** Validation for CRISPR/Cas9-mediated deletion of a splicing silencer motif. The splicing silencer motif in *POT1* disrupted by a mutation in MALY-4120193 (Fig. 5d bottom) was deleted by using CRISPR/Cas9 genome editing. This deletion was verified by Sanger sequencing. RT-PCR using primers spanning exons 6 and 7 showed cryptic exon activation, which was confirmed by Sanger sequencing.

We investigated the extent to which intronic mis-splicing mutations account for tumour suppressor gene disruptions. To this end, we compared the proportion of tumour samples with intronic mis-splicing mutations to those with truncating mutations (i.e., nonsense substitutions, frameshift indels and disruptions of the canonical SS dinucleotide) per known tumour suppressor gene (Fig. 5a). We found 64 tumour suppressors for which intronic mutations account for >5% of mutant tumour samples. The percentage of cancer samples with mis-splicing intronic mutations in these genes ranged up to 50% (Fig. 5a). Thirteen of the genes were affected by deep intronic mutations. Seven of the 64 tumour suppressors (*ACVR2A*, *CSMD3*, *EPS15*, *MAP2K4*, *NF1*, *PBRM1* and *RB1*) had intronic mis-splicing mutations in multiple tissues. Among these genes, *RB1* stood out with intronic mis-splicing mutations in five different cancer types (Fig. 5b). Among the tissue types analysed, lymphoid tissue type showed the highest proportion of deep intronic mutations (Supplementary Fig. 8), leading us to further investigation of known and potential tumour suppressors in this tissue type (Fig. 5c). For example, deep intronic mis-splicing mutations made up 50% of the inactivating somatic variants of *POT1* in lymphoid tissue. POT1 inactivation drives tumourigenesis by enhancing telomere replication stress and genomic instability [32, 33]. For one of the two pseudoexon activation events illustrated in Fig. 5d, we performed experimental validation by CRISPR/Cas9 genome editing (Fig. 5e). When the silencer motif containing the identified mutation was deleted, the insertion of the cryptic exon was observed at the RNA level. In addition to known suppressors (*CSMD3*, *EBF1*, *FBXO11*, *NFKBIE*, and *POT1*), potential suppressors are affected by mis-splicing intronic mutations. For example, *TMSB4X*, for which intronic mutations account for 67% of mutant samples, has recently been reported to be recurrently truncated in diffuse large B-cell lymphoma [34, 35]. Interestingly, the proximal mis-splicing mutations we identified in three different samples of the same tumour type were exactly the same substitution (Fig. 1d). Frequent loss-of-function mutations were also observed for *CD58* [36]*, IGLL5* [37]*, IRF1* [38]*, LTB* [39]*, MGA* [40] and *VMP1* [41] in blood cancers, implicating a potential tumour-suppressive role of these genes in the pathogenesis in this tissue type. Overall, these results indicate that intronic mis-splicing mutations are previously underappreciated mechanisms of cancer gene disruption.

## Discussion

Our analysis of paired WGS and RNA-seq data across a large number of tumour samples portrayed the genomic landscape of intronic mutations that alter mRNA splicing. The high validation rates of abnormal splicing by allele specificity ensured the reliability of the mutations that were identified by our read ratio-based method. Our comprehensive analysis identified a large set of mis-splicing mutations that (1) disrupt core splicing codes such as donor/acceptor SSs, PPTs, and BPs; (2) activate cryptic donor/acceptor SSs and PPTs; or (3) alter auxiliary splicing codes such as splicing enhancers and silencers. We characterised these mis-splicing mutations in detail and uncovered the signatures of positive or purifying selection in cancer on the transcripts carrying NMD-sensitive PTCs caused by aberrant splicing. In particular, we demonstrate that frequent gain of splicing enhancers or loss of splicing silencers by deep intronic mutations represents an important, previously unappreciated mechanism of partial intron retention and pseudoexon activation in human cancers. We experimentally validated the pseudoexon activation by deleting the silencer motif using CRISPR/Cas9 genome editing. Overall, deep intronic mutations comply with the GT-AG rule and mostly act through the modulation of splicing codes encompassing the SSs, PPTs, BPs, and splicing enhancers or silencers.

The current study substantially extends our knowledge on mis-splicing intronic mutations in human cancers. We found a large number of known tumour suppressors affected by intronic mutations. For example, the proportion of mutant samples affected by intronic mutations in tumour suppressors ranged up to 50% (e.g., *POT1*). We also found tumour suppressors affected by intronic mutations in multiple tissue types, such as *ACVR2A*, *CSMD3*, *EPS15*, *MAP2K4*, *NF1*, *PBRM1* and *RB1*. In addition, we identified a number of potential tumour suppressors affected by intronic mutations in lymphoid tissues where deep intronic mis-splicing mutations were frequently observed. For example, *TMSB4X*, for which intronic mutations accounted for 67% of mutant samples, had the same substitution in three different samples. This result suggests that somatic SNVs beyond intronic SSs should be carefully considered for their potential as disease-causing variants.

One of the clinical implications of this finding is the application of splice-switching antisense oligonucleotides (SSOs) as therapeutic drugs [42]. The hybridisation of SSOs to splicing regulatory elements results in the modification of mRNA splicing by several distinct mechanisms of action [43]. The most notable example of SSO therapy is the FDA-approved nusinersen, which targets an intronic splicing silencer of SMN2 and prevents truncated protein production by restoring its normal splicing [44]. Therefore, a systematic identification of splicing-alerting somatic mutations especially in TSGs holds considerable promise for cancer therapeutics.

This study highlights the importance of proximal as well as deep intronic somatic mutations in TSG inactivation by aberrant splicing. Mis-splicing mutations in proximal intronic regions accounted for a large fraction of cancer gene disruptions across different cancer types. In particular, deep intronic mis-splicing mutations showed a potency for TSG inactivation similar to that for exonic mis-splicing mutations. However, the number of deep intronic mutations, particularly in TSGs, may have been underestimated because PTC-mediated NMD decreases the number of detectable mRNA molecules. Therefore, the development of sequence-based predictive models for mis-splicing deep intronic mutations, as SpliceAI [45] which can predict alternative splicing from sequence, will provide valuable insights into cancer gene disruption. In this work, we constructed machine learning models for proximal intronic mutations, which are likely to prove beneficial as part of the standard analysis pipeline for cancer and other diseases. A larger number of WGS samples will be required to train learning models with a sufficient number of cases in which deep intronic mutations have altered splicing.

## Materials and methods

### Somatic mutation data

On the basis of called variants (MAF format) from WGS data generated by the PCAWG project, we first excluded tumour samples with known pathogenic mutations in *trans*-acting splicing factors [46], including *U2AF1* (exons 2 and 6), *SF3B1* (exons from 13 to 16), *SRSF2* (exon 1), and *ZRSR2* (all exons), to assess the direct effect of *cis*-acting mutations on mRNA splicing. We further excluded samples showing a higher fraction of reads spanning novel junctions, based on the assumption that those with deficiency in spliceosome machinery would have the higher fraction. Specifically, we computed the fraction of reads spanning novel junction, including partial (one of the junctions is known) and complete novel ones (both junctions are unknown), over all junction reads for each sample using RSeQC [47]. We then converted this fraction into *z*-score per cancer type and then eliminated samples for which *z*-score was >1.96 (i.e., *p* value < 0.05), resulting in 1134 samples. Somatic variants called by at least two different callers were included in further analysis. We used the DNA allele frequency of SNVs in the MAF file. We filtered out somatic mutations that overlapped with common single nucleotides polymorphisms (SNPs), of which the minor allele frequency (MAF) was >1% in dbSNP147 [48]. Those that were overlapped with other SNPs (MAF < 1% or unknown) were filtered out unless they were supported by at least two different tumour samples in COSMIC v81 (confirmed somatic variants) [49]. Called somatic structural variations (SVs) and copy number alterations (CNAs) were obtained from the PCAWG. The distance of SNVs from the nearest exon-intron junction was measured based on the longest transcript. We obtained whole-exome sequencing-based variant calls from TCGA data portal and selected variants for which coverage was >20.

### RNA-seq data and processing

We downloaded TopHat-based aligned [50] RNA-seq BAM files from the PCAWG. We extracted uniquely aligned RNA-seq reads near variants using Samtools [51] and discarded those marked as PCR duplicates or non-primarily alignments (or supplementary alignments). PCR duplicates of RNA-seq reads were marked using Picard. The reads that did and did not span known exon-exon junctions in GENCODE v19 Basic gene model [52] were classified as those supporting normal splicing and abnormal splicing, respectively. The RNA-allele frequency at SNV positions was computed on the basis of alleles with a base quality ≥30. For control samples, we obtained 3022 control normal RNA-seq BAM files (TopHat-based aligned) from two sources: (i) 2860 normal samples from the Genotype-Tissue Expression (GTEx) project [53]; and (ii) 162 normal samples from the PCAWG project. We used the same procedure to process the control RNA-seq BAM files as described above.

Gene expression data that were normalised with the FPKM-upper quantile method were obtained from the PCAWG. For each gene per cancer type, the control mRNA expression was calculated by averaging the gene expression levels of wild-type samples without non-silent mutations, including translocations and other types of SVs with breakpoints, and CNAs (medium confidence). At least five wild-type samples were required to calculate control mRNA expression.

### Ratio-based splicing analysis

The ratio-based splicing analysis detected somatic mutations that were associated with six types of abnormal splicing: full and partial intron retention, full and partial exon skipping, pseudoexon activation, and combinatorial abnormal splicing that showed multiple splicing aberrations. SNV-causing full intron retention was required to be confirmed only at the allele level (see below). To assess the direct effect of intronic mutations on mRNA splicing, we filtered out mutations having additional variants nearby (details given in Supplementary Fig. 2), or non-silent mutations (missense, nonsense, exonic indels and SSs variants) including translocations and other types of SVs with breakpoints within exons in a given entire gene. Silent SNVs within the vicinity of exon-intron junctions (i.e., exonic SNVs within 2 bp of junctions) were categorised as non-silent mutations. When assessing the effect of non-silent mutations, genes with multiple non-silent mutations were excluded.

Mutations within a 30 bp-flanking region of exon-intron junctions or of activated intronic cryptic SSs were subjected to the ratio-based splicing analysis. Intronic mutations were classified into the following three categories according to distance to exon-intron junction: those within 1–2 bp, 3–20 bp, and >20 bp regions from the junction were defined as SSs, proximal, and deep intronic mutations, respectively. For deep intronic mutations, we limited our analysis to those surrounding BPs (known/predicted BPs and 5 bp upstream and 3 bp downstream of the BPs) or activated cryptic SSs (30 bp-flanking region of cryptic SSs) associated with partial intron retention and/or pseudoexon activation to ascertain the relevance of their effect on abnormal splicing.

According to the defined ratio criteria for each type of abnormal splicing (details in Supplementary Fig. 2), we first calculated the proportion of abnormally spliced reads to total reads (sum of normally and abnormally spliced reads). For the unbiased calculation, we required the number of both types of the spliced reads ≥3 for each abnormal splicing event. Next, we assessed whether the proportion was significantly higher than expected given the background distributions estimated from the control normal and cancer samples. For each event, the background distribution was derived separately from the normal and cancer samples with ≥3 normally spliced reads (example distributions are shown in Fig. 1c). For the accurate estimation of background distributions, we excluded tumour samples with non-silent mutations including translocations and other types of SVs with breakpoints within exons in a given gene, and required more than 500 samples for generating each of the two distributions. Based on the two distributions, the observed proportion was then transformed into the Z-score to estimate its *P* value (right-tailed). For a somatic mutation to be associated with abnormal splicing, we required its observed proportion to be within the top 1% of both normal and cancer sample-based distributions and its FDR < 0.1. Since our analysis detected abnormal splicing based on local exon-intron structure, when the same abnormal splicing event was observed in multiple isoforms, we selected the longest one for further analysis.

### Validation by allele specificity

To assess the reliability of the identified SNVs through the ratio-based splicing analysis, we validated them in an allele-specific fashion. For abnormal splicing associated with cryptic site activation (partial intron retention, pseudoexon activation and/or partial exon skipping), we assessed the extent to which the RNA-seq reads supporting cryptic splice site activation were variant-specific. For this, we computed odds ratios from the 2 × 2 contingency table of the number of reads with columns indicating the reference and variant allele and rows denoting the DNA and RNA allele. The RNA allele count at an SNV position was derived from the RNA-seq reads showing cryptic site activation. Among the SNVs within a 30 bp-flanking region of activated cryptic splices sites, those spanned by the RNA-seq reads showing cryptic splice site activation (number of reads ≥3) were defined as verifiable SNVs. Those with an odds ratio >1 were classified as showing variant-specific abnormal splicing. For exon skipping, exonic SNVs on skipped exons are expected to show reference-allele specific expression. Thus, we assessed the extent to which exonic SNVs causing exon skipping showed reference allele-specific expression, using the odds ratio from the 2 × 2 contingency table of the number of reads with columns indicating the reference and variant allele and rows denoting the DNA and RNA allele. The DNA and RNA allele counts were derived from the DNA- and RNA-seq reads spanning the SNV. Exonic silent or missense SNVs causing exon skipping were defined as verifiable SNVs. Those with an odds ratio <1 were classified as showing reference allele-specific expression.

### Identification of intronic mis-splicing SNVs by allele specificity

#### Confirmation of full intron retention at the allele level

Full intron retention by intronic SNVs that was identified through ratio-based splicing analysis was required to show variant-allele-specific expression. Among those with an odds ratio > 1, we further selected those SNVs where the number of the variant allele reads was ≥3 and the fraction of the variant alleles was >80%. For exonic SNVs, we exploited the 2 × 2 contingency table of the number of reads with columns indicating the reference and variant alleles from RNA and rows denoting the reads spanning normal exon-exon junctions (spliced) and exon-intron junctions (unspliced). The un-spliced reads (number of reads with variant allele ≥3) were required to cover 5 bp of both the exon and intron regions. We performed the Fisher’s exact test on the contingency table, and SNVs with FDR of <0.1 were included for further analysis.

#### Rescue of intronic SNVs excluded in the ratio-based analysis

We leveraged allele-level information to rescue deep intronic SNVs on partially retained intron (and/or pseudoexon) excluded in the ratio-based splicing analysis due to its rigorous criteria (Supplementary Fig. 9). For the SNVs excluded due to insufficient breadth of coverage in introns, we selected SNVs where the variant allele-specific abnormally spliced reads (≥3) relative to the total abnormally spliced reads was >80% (*n* = 20; examples in Supplementary Fig. 10; Supplementary Table 4b). For the other SNVs excluded due to their distance from cryptic SSs (>30 bp), those showing variant allele-specific splicing (example in Supplementary Fig. 11a) or variant allele-specific expression within 100 bp of cryptic SSs (example in Supplementary Fig. 11b) were included by applying the same criteria above (*n* = 22; Supplementary Table 4c).

### Splicing strength estimation and branchpoint annotation

The MAXENT tool [27] was used to estimate the donor and acceptor splicing strength of mis-splicing SNVs with the wild-type or mutant allele. Aside from SNVs at canonical dinucleotides (GT and AG), SNVs at positions from −3 to 6 and from −3 to 20 near the donor and acceptor SSs were used to calculate the donor and acceptor splicing strengths, respectively. Among the identified mis-splicing mutations between 15 and 60 bp from authentic acceptor splice junctions, we annotated them to the BPs and their flanking residues (i.e., 5 bp upstream and 3 bp downstream of the BPs) using the following resources: supplementary table 1 from Mercer et al. [4], Taggart et al. [54], and the predicted list from Signal et al. [55]. We selected the closest BPs when there were multiple annotated BPs near the mutations. The binding free energy of the *U2* snRNA motif *(GUGUAGUA)* to mis-splicing SNVs in the flanking residues of the BPs were estimated using the RNAduplex script from Vienna RNA package [56]. Motif logos were generated using WebLogo [57].

### Enrichment test for splicing enhancers, silencers, and cancer genes

We obtained previously defined exonic splicing enhancer and silencer hexamer motifs from the following publications: enhancers from Ke et al. [58] (*n* = 400) and Fairbrother et al. [59] (*n* = 238) and silencers from Ke et al. [58] (*n* = 400), Wang et al. [60] (*n* = 176), and Wang et al. [61] (*n* = 82; because intronic enhancers function as exonic silencers). Both the 5 bp upstream and downstream flanking sequences of deep intronic mis-splicing SNVs on pseudoexons (and/or partially retained introns), except for those lying within the core SS code (−3 to −1), were extracted. We then compared the sequences with the mutant and wild-type allele of the SNVs against the motifs in each set to assess the degree of consensus. If a given sequence overlapped with multiple motifs, the sequence was counted only once. To test whether the observed number of overlap (*N*) was greater than expected, we generated a null distribution by randomly selecting the same number of SNVs from all deep intronic SNVs (intronic positions >20 from authentic junctions) 100,000 times. The enrichment *P* value was then calculated as the proportion of random permutations in which the overlap count was greater than or equal to the actual observed overlap count (*N*). The union of motifs for each enhancer and silencer was compared against the given sequences in Figs. 1g and d. The enrichment of exonic SNVs causing exon skipping (*n* = 91) in enhancers and silencers was performed as above (Supplementary Fig. 3c) (a random selection of 91 exonic SNVs at exonic positions −30 to −4, repeated 100,000 times).

Cancer driver genes commonly altered by splice site mutations were derived from COSMIC v81 census (*n* = 76; marked as ‘S’ in the mutation type column) [49]. Previously defined core and extended driver tumour suppressor and oncogene genes were taken from the following publications: 71 and 300 tumour suppressors from Vogelstein et al. [62] and Davoli et al. [63], and 54 and 250 oncogenes from Vogelstein et al. [62] and Davoli et al. [63], respectively. A total of 1878 essential genes were derived from the CRISPR-based screen [64], and those overlapped with the tumour suppressors or oncogenes were discarded. We compared the mis-splicing SNVs against each cancer gene set and then assessed whether the observed count was greater than expected using the random permutation described above. A null distribution was generated by randomly picking the same number of SNVs 100,000 times.

To identify more tumour samples harbouring genes disrupted by intronic cryptic site activation associated with partial intron retention/pseudoexon activation (Fig. 5a–c), We also included the mutations that were excluded because of redundant mutations within the same intron when the mutant allele was specifically carried in mis-spliced reads (Supplementary Table 11). Specifically, mutations passing the first criteria of partial intron retention and/or pseudoexon activation in ratio-based splicing analysis and showing allele-specific splicing pattern were included.

### PTC and NMD analysis

We examined whether abnormally spliced transcripts harboured a PTC. We first selected the longest isoform when there were multiple isoforms showing the same abnormal splicing event (because our analysis detected abnormal splicing based on local exon-intron structure). For SNVs inducing multiple forms of the same type of abnormal splicing (e.g., partial intron retention with different activated cryptic SSs; example in Fig. 1e), we chose the form with the highest proportion rate (i.e., abnormally spliced reads relative to total reads) for checking the presence of PTC. In the cases when an individual event in the complex combinatorial splicing resulted in inconsistent PTC outcomes (i.e., PTC presence or absence and NMD eliciting or escaping under the presence of PTC), SNVs causing this type of abnormal splicing were not included in this analysis (Fig. 4c, d). In addition, SNVs that did not affect coding exon splicing (e.g., SNVs altering UTR splicing) were not included. The PTC-generating SNVs were further divided into NMD-sensitive and NMD-insensitive PTCs. PTCs within the first 250 bp or between 55 bp upstream of the last exon-exon junction and the end of the last exon were classified as NMD-insensitive PTCs [31], while the remaining were classified as NMD-sensitive PTCs.

### Prediction models

One-class SVM with the linear kernel model [65] was used to build classifiers to discern mis-splicing SNVs. We used the python scikit-learn package with default parameters, except for the nu parameter (an upper bound on the fraction of training errors and a lower bound on the fraction of support vectors), which was set to 0.05. We built the three binary classifiers based on the identified mis-splicing SNVs at (i) donor intronic (*n* = 154; positions from 3 to 6), (ii) donor exonic (*n* = 110; positions from −2 to −1), and (iii) acceptor intronic (*n* = 30; position 3) positions. Each model was trained with two features of splicing strength differences between the mutant- and wild-type allele of the mis-splicing SNVs computed from MAXENT; the models for SNVs at the donor SSs (i and ii) used 5′splice_MUT_ – 5′splice_WT_ and of 5′splice_MUT_ – 3′splice_WT_, and the model for the acceptor SSs (iii) used 3′splice_MUT_ – 3′splice_WT_ and of 3′splice_MUT_ – 5′splice_WT._ The classification performance was evaluated with 1000 iterations of threefold cross-validation. For accuracy comparison, we trained models by randomly selecting the same number of SNVs (100,000 times) from all SNVs at corresponding sites. We then assessed the performance with threefold cross-validation. The prediction models have been fully implemented as a publicly available python script.

### CRISPR/Cas9-mediated genome editing

A guide RNA (sgRNA) flanking the *POT1* target site (chr7:124,532,129) was designed by RGENs (http://www.rgenome.net). This sgRNAs was cloned into pSpCas9(BB)-2A-GFP (PX458, Addgene, #48138). The pSpCas9(BB)-2A-GFP vector with sgRNA was transfected into K562 cells using the Neon® Transfection System Kit (Thermo Fisher Scientific). After 48 h, the transfected GFP-positive cells were individually isolated. The single-cell clones were individually cultured in single wells for 2 weeks. To verify the deletion of the *POT1* target site, genomic DNA was extracted from the transfected cells by the DNeasy Blood and Tissue Kit (Qiagen), and RNA was extracted by the RNeasy Plus Mini Kit (QIAGEN). cDNA was synthesised from total RNA using SuperScript IV VILO Master Mix (Invitrogen) and amplified by PCR using Hifi Hot Start (KAPA). The obtained PCR products were sequenced in both the forward and reverse orientations by Sanger sequencing. All of the PCR primers and CRISPR sgRNA sequences are provided in Supplementary Table 12. We used the K562 (CCL-243) cell line, which was obtained from the American Type Culture Collection. This cell line was cultured in complete RPMI-1640 medium (Life Technologies) supplemented with 10% foetal bovine serum (Life Technologies) and 1% penicillin-streptomycin (Life Technologies). The cells were maintained at 37 °C in a humidified chamber supplemented with 5% CO2.

## Supplementary information

Supplementary Figures

Supplementary Information

Supplementary Tables

## Data Availability

Codes for the prediction of proximal intronic mis-splicing mutations, https://github.com/kaistomics/splicing.
