# Supplementary material for: Comprehensive characterisation of intronic mis-splicing mutations in human cancers
Source: Oncogene. 2021 Jan 8;40(7):1347–61. doi: 10.1038/s41388-020-01614-3 (PMC7892346; doi:10.1038/s41388-020-01614-3)

Supplementary figure 1

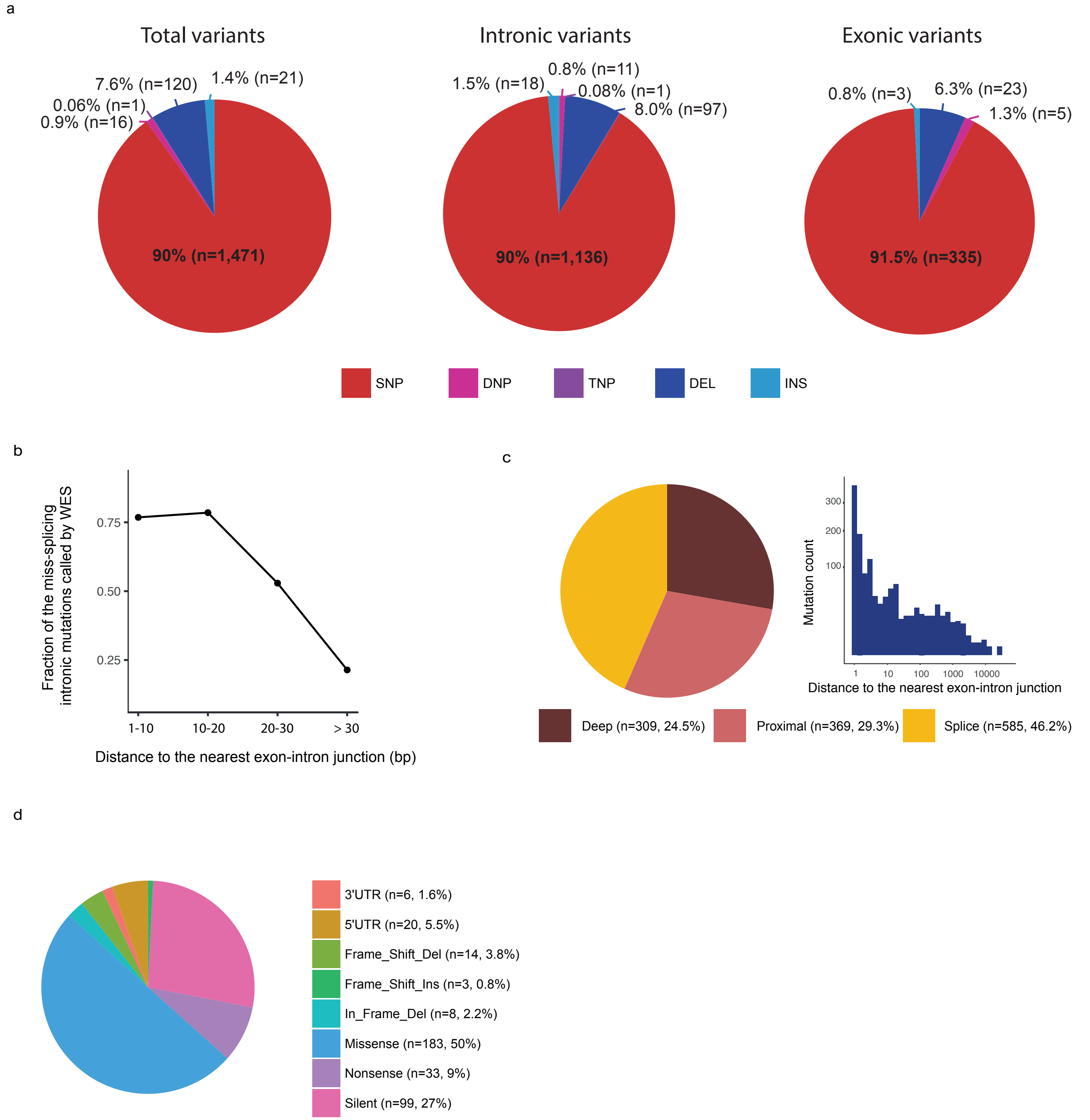

Supplementary figure 2

Abnormal splicing type	Schematic representation	Criteria

a Partial intron retention

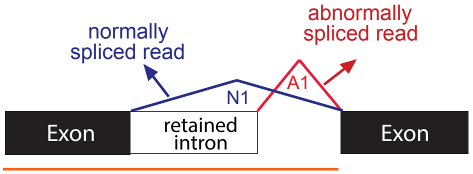

① $\frac{A1}{A1 + N1}$

② breadth of coverage in retained intron > 80%

b Pseudo exon activation

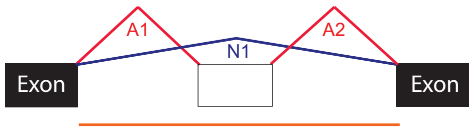

①-1 $\frac{A1}{A1 + N1}$ ①-2 $\frac{A2}{A2 + N1}$

② breadth of coverage in retained intron > 80%

c Full intron retention

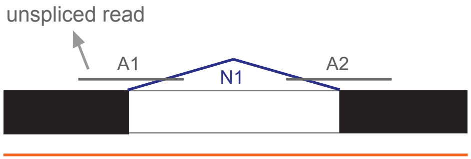

① $\frac{A1}{A1 + N1}$ ② $\frac{A2}{A2 + N1}$ ③ breadth of coverage in retained intron > 80%

④-1 Intronic SNV

fraction of variant allele expression > 80%

④-2 Exonic SNV

	ref	alt
N1		
A1 (or A2)		

p value from fisher's exact test < 0.1

d Partial exon skipping

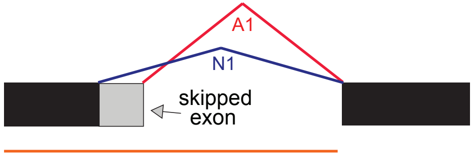

① $\frac{A1}{A1 + N1}$

e Full exon skipping

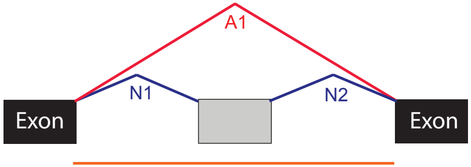

① $\frac{A1}{A1 + N1 + N2}$

② coverage of skipped exon < average coverage of the adjacent exons

Supplementary figure 3

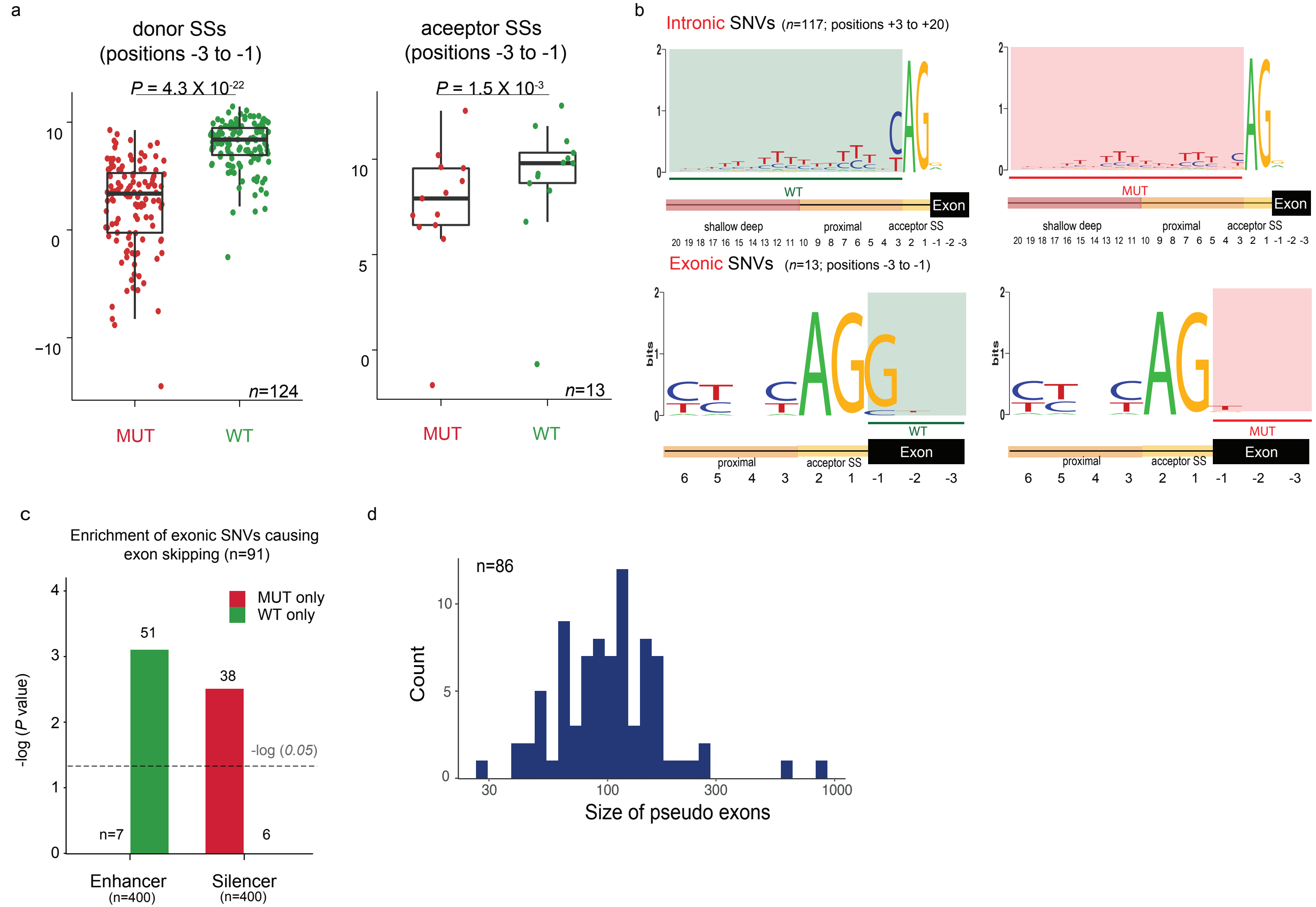

Supplementary figure 4

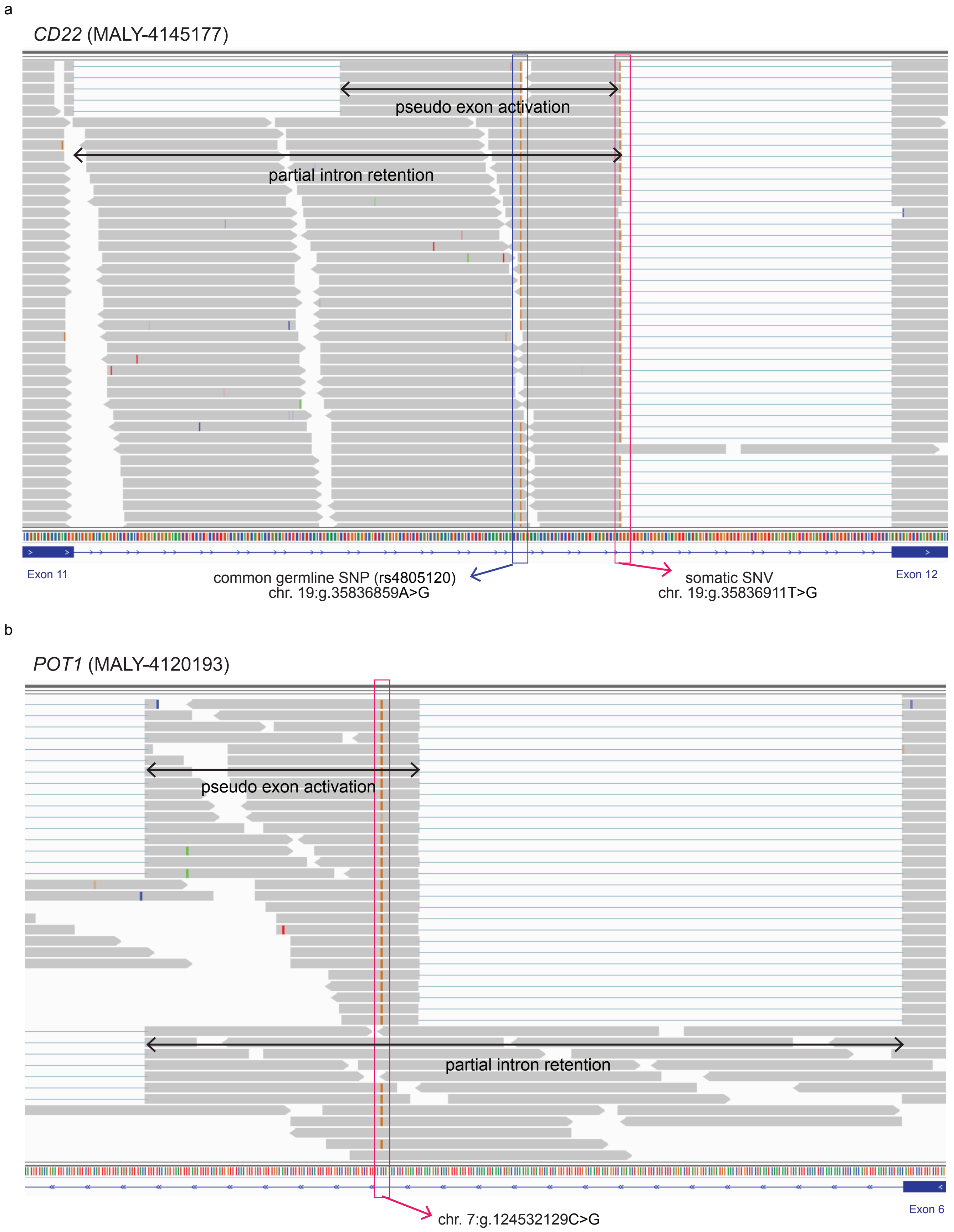

Supplementary figure 5

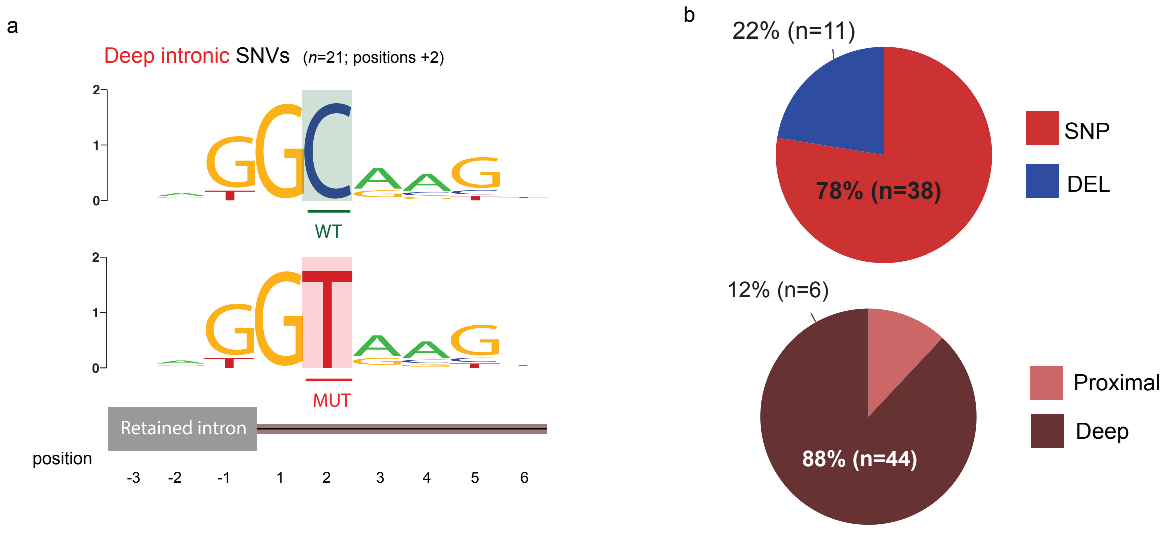

Supplementary figure 6

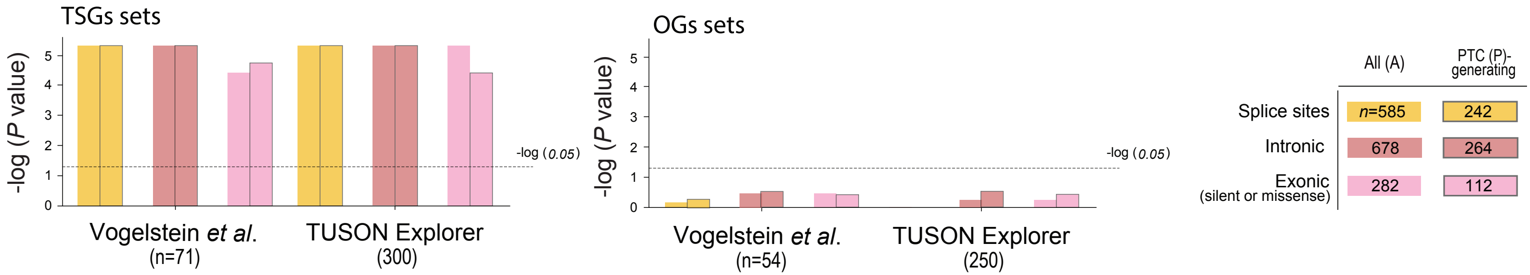

Supplementary figure 7

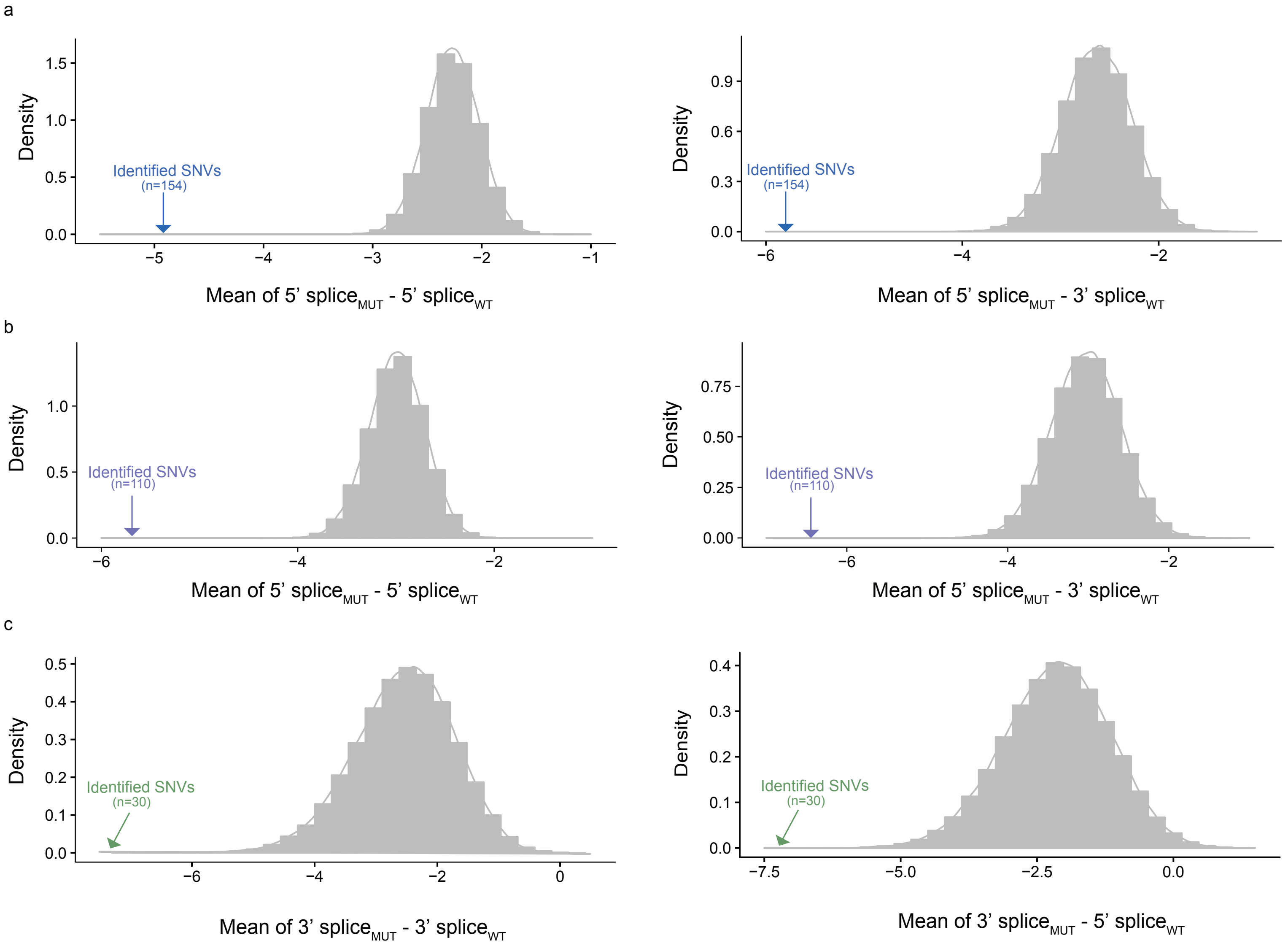

Supplementary figure 8

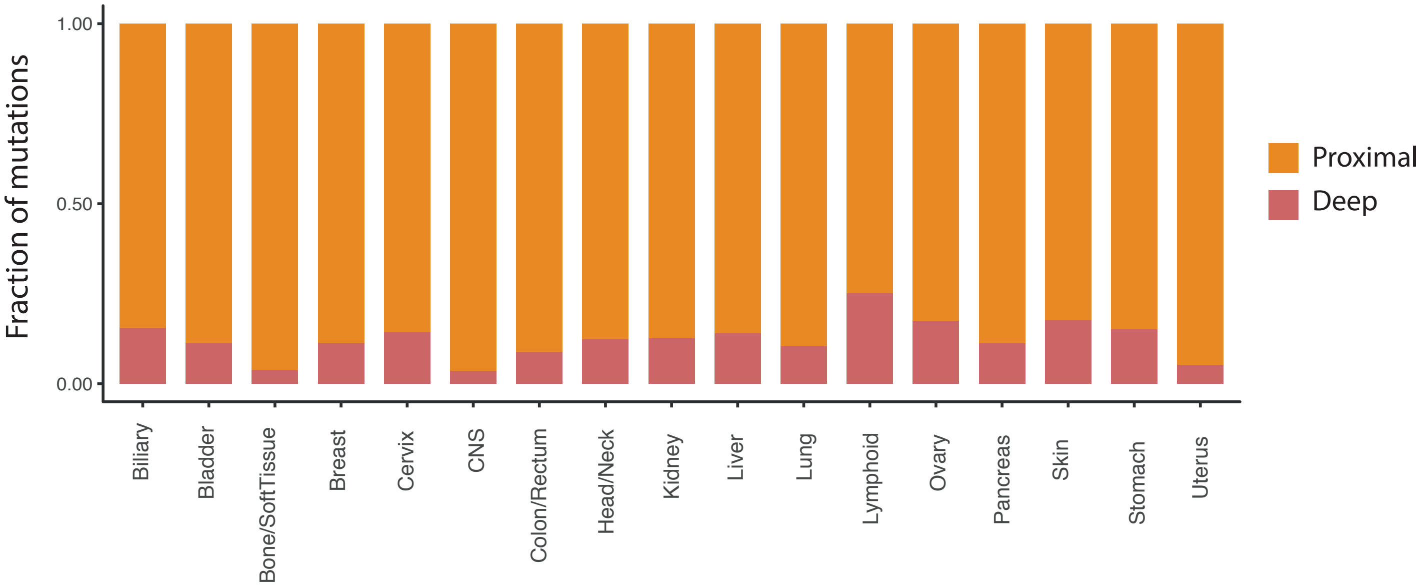

Supplementary figure 9

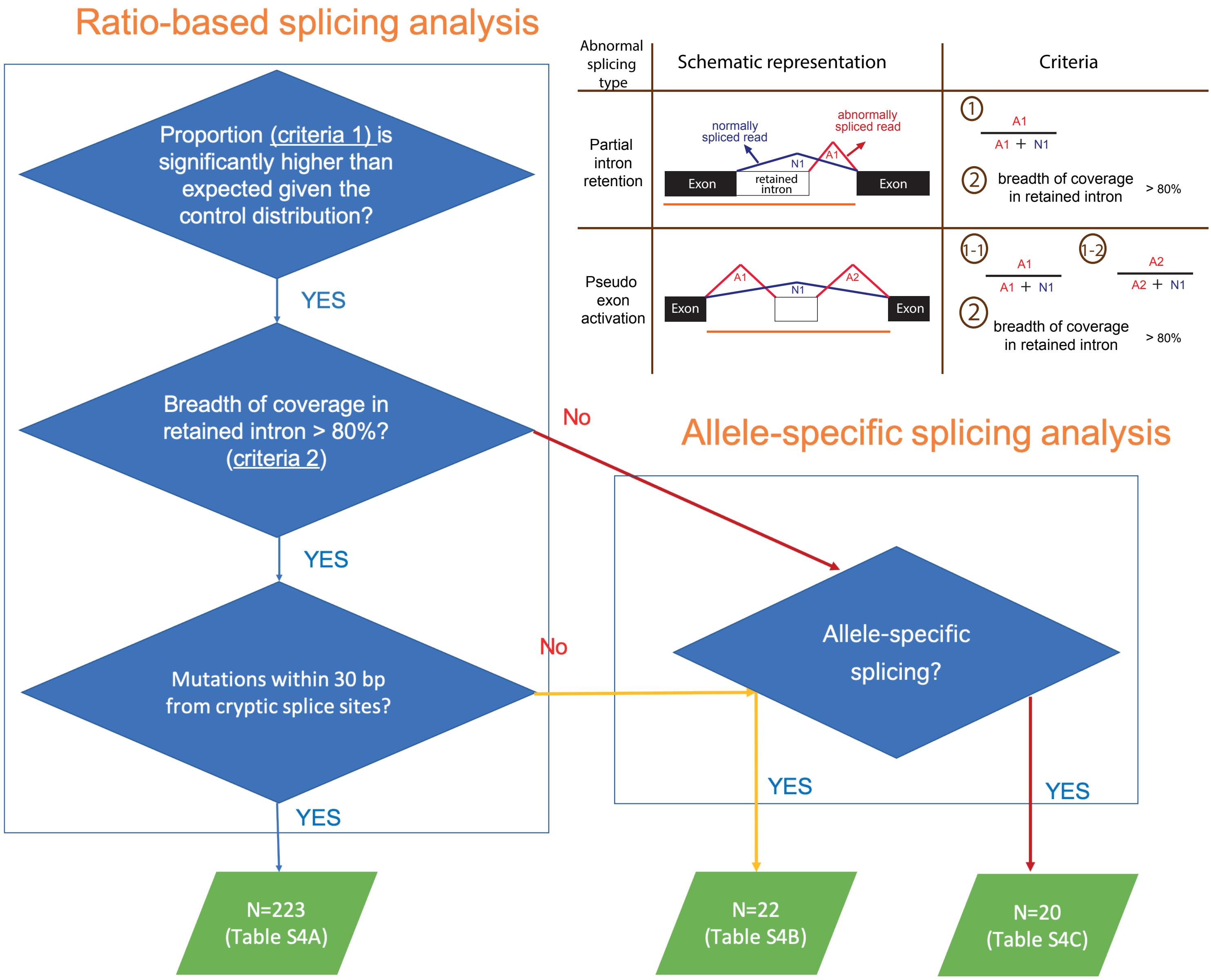

Supplementary figure 10

a

MBP (TCGA-05-4389)

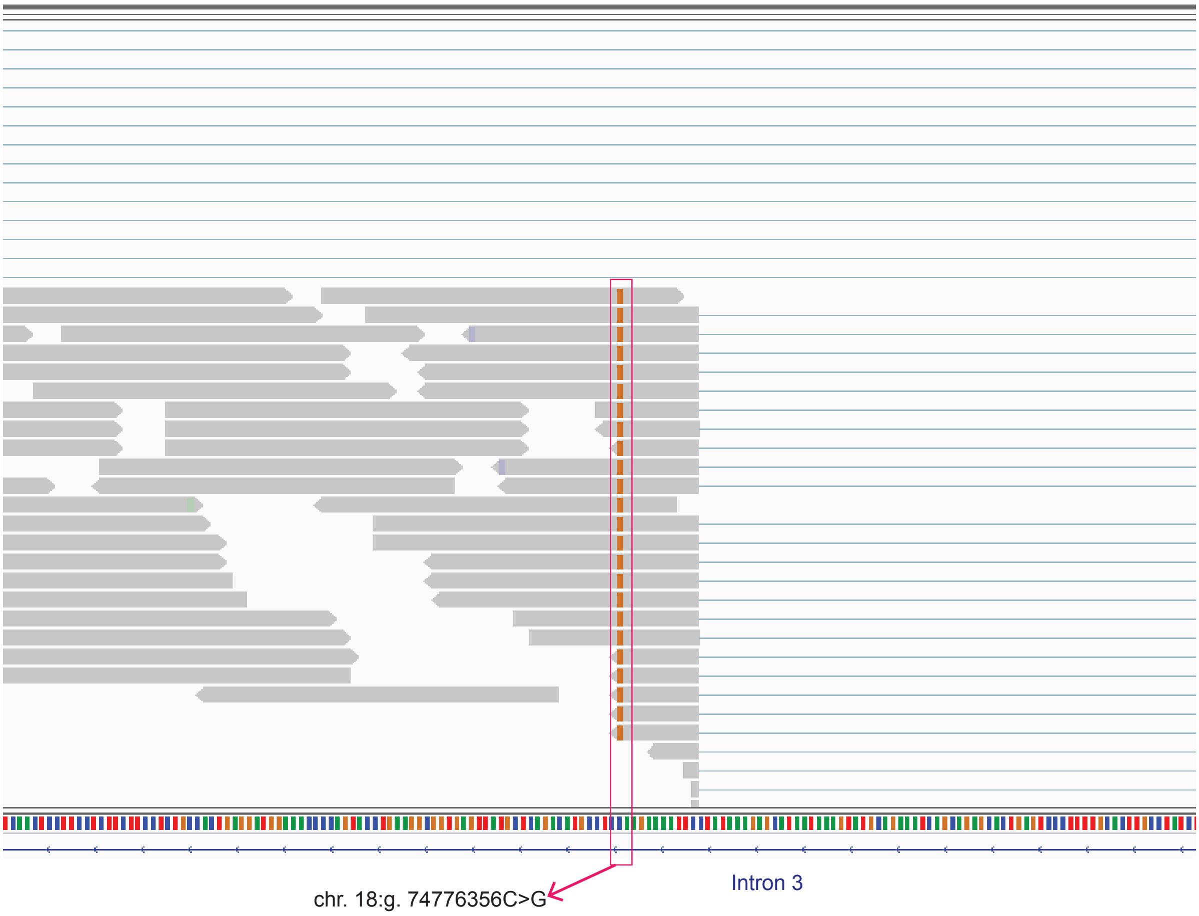

b

ADORA2B (TCGA-F1-6875)

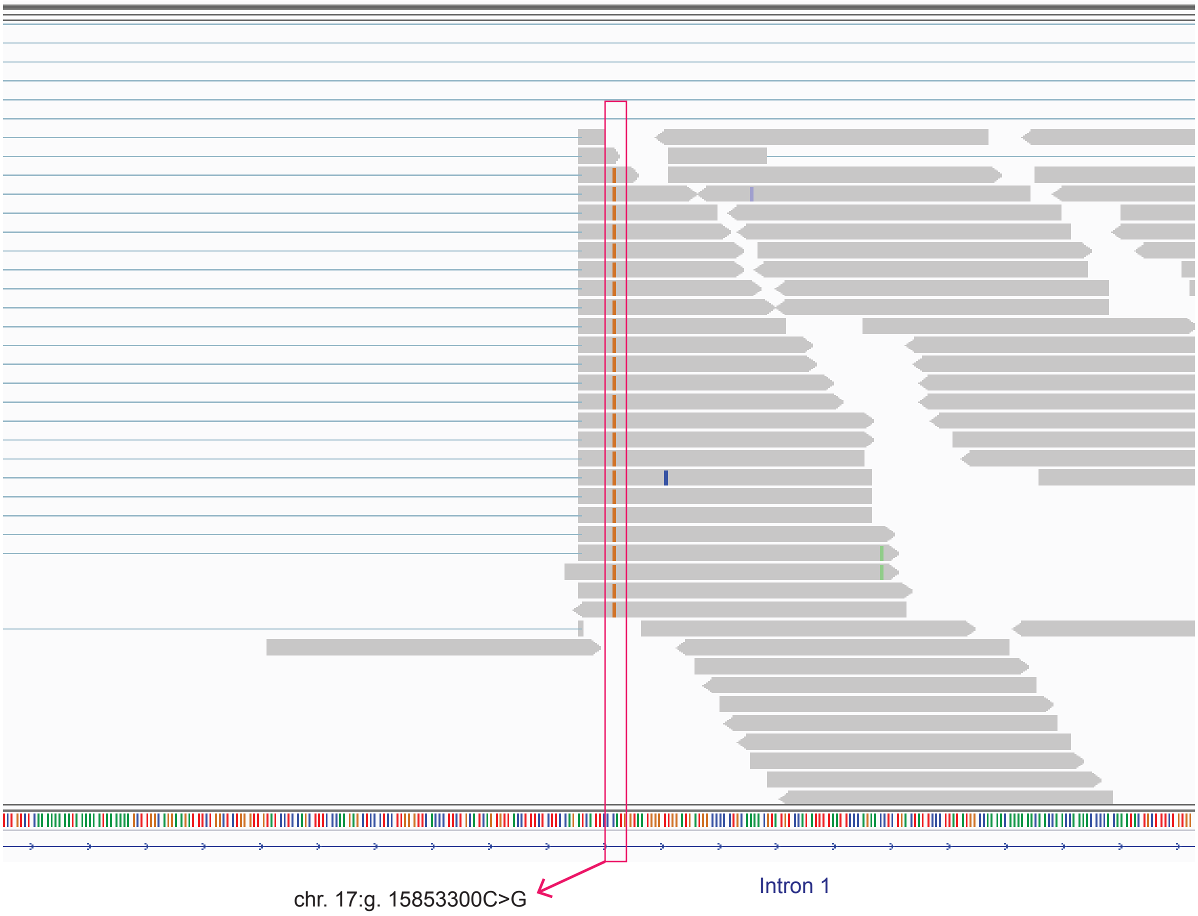

Supplementary figure 11

a

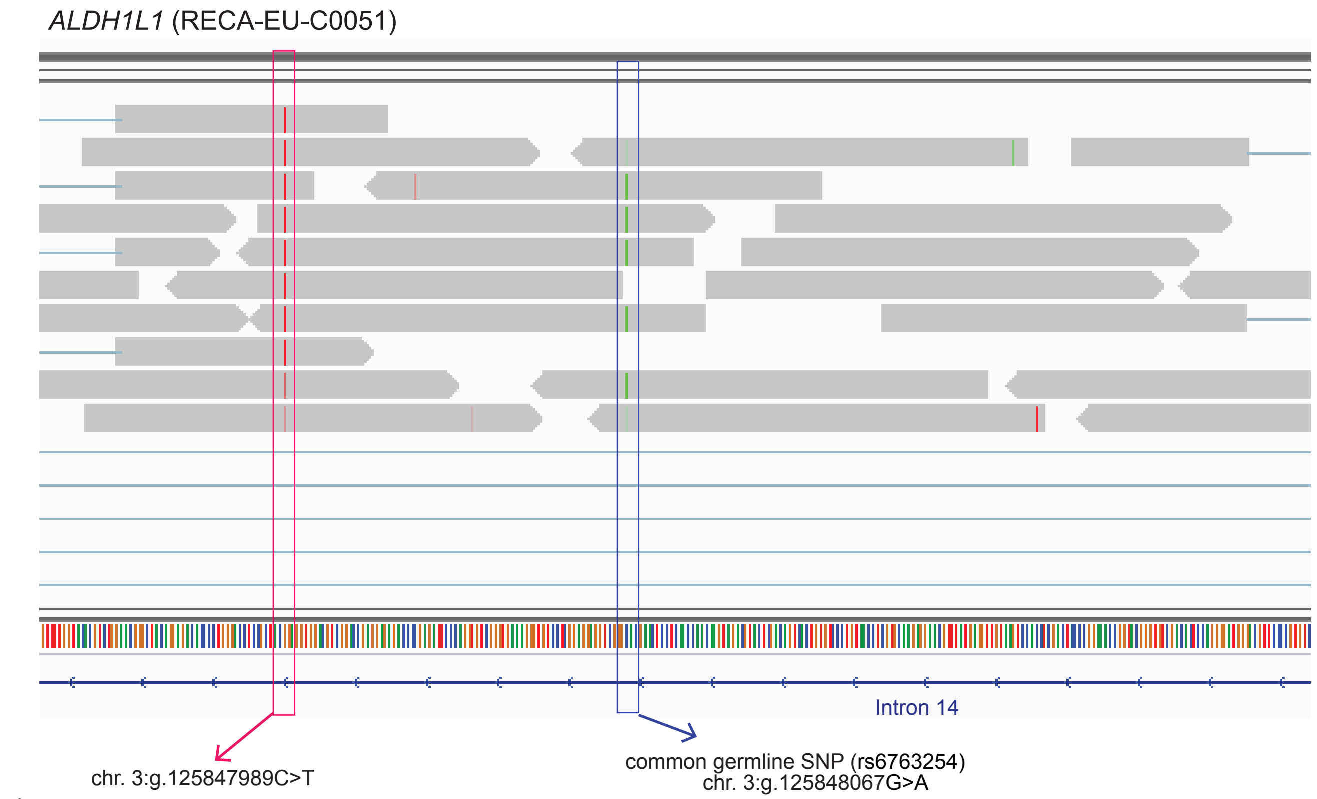

b

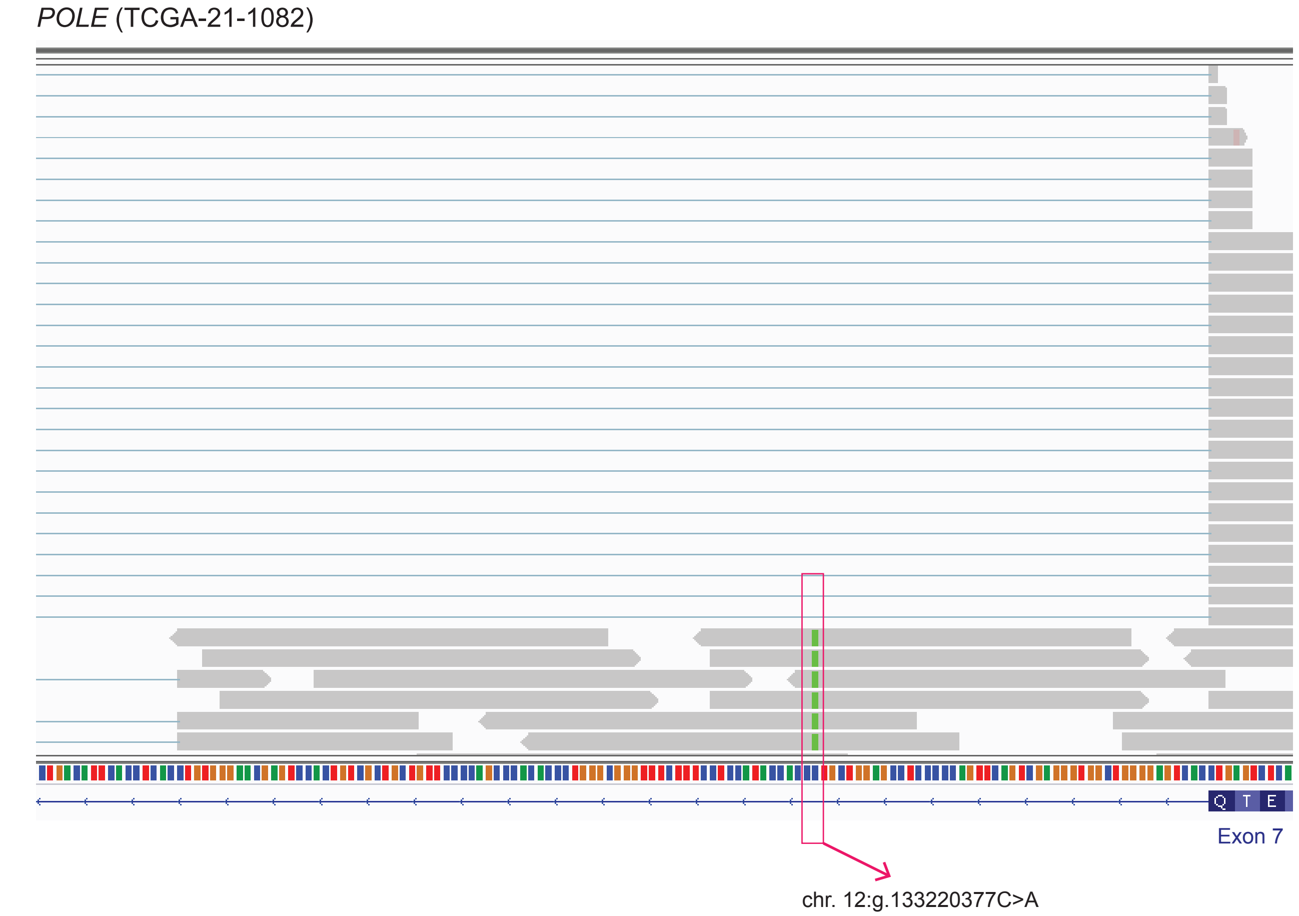

Supplement: Supplementary file 1 — Supplementary Figures [file 41388_2020_1614_MOESM1_ESM.pdf]
