# Supplementary material for: Comprehensive characterisation of intronic mis-splicing mutations in human cancers
Source: Oncogene. 2021 Jan 8;40(7):1347–61. doi: 10.1038/s41388-020-01614-3 (PMC7892346; doi:10.1038/s41388-020-01614-3)
Supplement: Supplementary file 2 — Supplementary Information [file 41388_2020_1614_MOESM2_ESM.docx]

Comprehensive characterization of intronic mis-splicing mutations in human cancers

Hyunchul Jung^1,2^*, Kang Seon Lee^1^* & Jung Kyoon Choi^1,3^

^1^Department of Bio and Brain Engineering, KAIST, Daejeon 34141, Republic of Korea

^2^Cancer Ageing and Somatic Mutation Programme, Wellcome Sanger Institute, Cambridge, UK

^3^Penta Medix Co., Ltd., Seongnam-si, Gyeongi-do 13449, Republic of Korea

*These authors contributed equally to this work.

Correspondence should be addressed to HJ ([hj6@sanger.ac.uk](mailto:hj6@sanger.ac.uk)) or JKC (jungkyoon@kaist.ac.kr).

**Supplementary Figure Legends**

**Supplementary Fig. 1.** Summary of the identified mis-splicing mutations. (A) Distribution of variant types. (B) Fraction of the miss-splicing intronic mutations called by whole-exome sequencing according to a distance to the nearest exon-intron junction. (C) Classification of intronic variants by their location and variant count as a function of the distance to the nearest exon-intron junction (C) Distribution of exonic variant types.

**Supplementary Fig. 2.** Detection criteria for five types of abnormal splicing. (A-E) The ratio-based splicing analysis was applied to mutations in the regions marked in orange where other variants (point mutations, indels, or breakpoints of structural variations) were absent. RNA-seq reads supporting abnormal and normal splicing were shown in red and blue, respectively. Normally spliced reads were defined as spanning known junctions in the GENCODE v19 basic gene model. Retained introns and skipped exons were represented as white and grey boxes, respectively. For full intron retention, unspliced reads (grey) covering at least 5 bp of both the exon and intron were counted.

**Supplementary Fig. 3.** (A) Differences in the strength of authentic donor and acceptor SSs between the mutant and wild-type allele. The Wilcoxon signed-rank test was used to infer the statistical significance of the differences. (B) Changes in sequence consensus by proximal intronic or exonic mutations near acceptor SSs. The consensus motifs were derived from sequences 18-bp upstream to 3-bp downstream of the AG dinucleotide. (C) Enrichment of exonic mutations causing exon skipping in the splicing enhancers and silencers. P values were calculated by random permutation tests. (D) Size distribution of the activated pseudoexons we identified.

**Supplementary Fig. 4.** Examples of the co-occurrence of pseudoexon activation and partial intron retention. IGV screenshots for mutations causing abnormal splicing in (A) *CD22* and (B) *POT1*.

**Supplementary Fig. 5.** (A) Distinct consensus motifs of mis-splicing mutations at cryptic donor SSs. (B) Percentage of variants near BPs categorized by the variant type (left) and location (right).

**Supplementary Fig. 6.** Enrichment of mis-splicing mutations in TSGs and oncogenes (OGs). The enrichment bars for PTC-generating mutations are bordered. P values were calculated by random permutation tests.

**Supplementary Fig. 7.** Distribution of the feature values of the prediction models. The density plots show the mean of feature values used in the model for (A) intronic mutations near donor SSs (+3 to +6), (B) exonic mutations near donor SS (-1 and -2), and (C) intronic mutations near acceptor SSs (-3). The distribution of expected values was obtained from random mutation selection (100,000 trials).

**Supplementary Fig. 8.** Proportion of proximal and deep intronic mutations per tissue type. The predicted proximal mutations were included.

**Supplementary Fig. 9.** Flowchart for rescuing mutations associated with partial intron retention and/or pseudo exon activation using allele-specific splicing analysis.

**Supplementary Fig. 10.** Examples of the rescued mutations causing intronic cryptic site activation. (A) *MBP* in TCGA-05-4389. (B) *ADORA2B* in TCGA-F1-6875.

**Supplementary Fig. 11.** Examples of the rescued mutations that were excluded in the ratio-based identification process due the distance between the mutation and cryptic SS ( > 30 bp). (A) *ALDH1L1* (A; allele-specific splicing) in RECA-EU-C0051. (B) *POLE* (B; allele-specific expression) in TCGA-21-1082.
